# A pilot study of sensory feedback by transcutaneous electrical nerve stimulation to improve manipulation deficit caused by severe sensory loss after stroke

**DOI:** 10.1186/1743-0003-10-55

**Published:** 2013-06-13

**Authors:** Kahori Kita, Yohei Otaka, Kotaro Takeda, Sachiko Sakata, Junichi Ushiba, Kunitsugu Kondo, Meigen Liu, Rieko Osu

**Affiliations:** 1 Department of Motor Control and Rehabilitation, ATR Computational Neuroscience Laboratories, Kyoto, Japan; 2 Department of Rehabilitation Medicine, Keio University School of Medicine, Tokyo, Japan; 3 Tokyo Bay Rehabilitation Hospital, Narashino, Japan; 4 Clinical Research Center, National Hospital Organization Murayama Medical Center, Musashimurayama, Japan; 5 Department of Biosciences and Informatics, Keio University, Yokohama, Japan

**Keywords:** Sensory feedback, Stroke rehabilitation, Sensory loss, Transcutaneous electrical nerve stimulation, Manipulation capability

## Abstract

**Background:**

Sensory disturbance is common following stroke and can exacerbate functional deficits, even in patients with relatively good motor function. In particular, loss of appropriate sensory feedback in severe sensory loss impairs manipulation capability. We hypothesized that task-oriented training with sensory feedback assistance would improve manipulation capability even without sensory pathway recovery.

**Methods:**

We developed a system that provides sensory feedback by transcutaneous electrical nerve stimulation (SENS) for patients with sensory loss, and investigated the feasibility of the system in a stroke patient with severe sensory impairment and mild motor deficit. The electrical current was modulated by the force exerted by the fingertips so as to allow the patient to identify the intensity. The patient had severe sensory loss due to a right thalamic hemorrhage suffered 27 months prior to participation in the study. The patient first practiced a cylindrical grasp task with SENS for 1 hour daily over 29 days. Pressure information from the affected thumb was fed back to the unaffected shoulder. The same patient practiced a tip pinch task with SENS for 1 hour daily over 4 days. Pressure information from the affected thumb and index finger was fed back to the unaffected and affected shoulders, respectively. We assessed the feasibility of SENS and examined the improvement of manipulation capability after training with SENS.

**Results:**

The fluctuation in fingertip force during the cylindrical grasp task gradually decreased as the training progressed. The patient was able to maintain a stable grip force after training, even without SENS. Pressure exerted by the tip pinch of the affected hand was unstable before intervention with SENS compared with that of the unaffected hand. However, they were similar to each other immediately after SENS was initiated, suggesting that the somatosensory information improved tip pinch performance. The patient’s manipulation capability assessed by the Box and Block Test score improved through SENS intervention and was partly maintained after SENS was removed, until at least 7 months after the intervention. The sensory test score, however, showed no recovery after intervention.

**Conclusions:**

We conclude that the proposed system would be useful in the rehabilitation of patients with sensory loss.

## Background

There are 3.2 million stroke patients in Japan and this number is continually increasing [1]. The most common impairments after stroke are motor deficits such as hemiparesis, which is experienced by more than 80% of stroke survivors [2]. Deficits in sensory abilities, on the other hand, are variously reported to be 11-85% [3], 65% [4], or 85% [5]. This variability is due to differences in assessment and the definition of sensory impairment. Severe sensory loss in the hand sometimes inhibits the patient’s ability to manipulate an object during daily activities, even when they have good overall motor function [6]. As a result of motor and sensory deficits, about 40% of patients lose use of the arm [2]. A great deal of time and effort is required to rehabilitate the affected limb and patients are often very focused on recovering as much arm function as possible once they have regained some mobility.

Sensory function, as well as motor function, is important for dexterity. Proprioceptive and haptic feedback, as well as vision, contributes to the learning and control of movements necessary to achieve a given task [7]. Various passive stimulation approaches have been tested in an attempt to regain lost sensory function, and as a result, recover motor function including: electrical stimulation, such as neuromuscular stimulation [8], cutaneous electrical stimulation [9,10], transcutaneous electrical nerve stimulation [11], intermittent pneumatic compression [12], thermal stimulation [13], and peripheral magnetic stimulation [14]. However, these techniques were limited to improving tactile and kinesthetic sensation. There is insufficient evidence to support the efficacy of these intervention strategies in improving manipulation capability [6].

It is widely recognized that, for improvement of manipulation capability, it is important to practice specific tasks in addition to training for general improvement of muscle strength, range of motion, etc. Task-specific training requires simply practicing the task and is effective for patients whose arm function is adequate to perform the task [15-18]. According to this view, to improve manipulation capability of patients with sensory loss, active training to perform object manipulation would be important rather than, or in addition to passive stimulation to recover sensation. Providing sensory information through alternate pathways during manipulation tasks has the potential to improve a patient’s dexterity, even though the original sensory pathway does not recover. Several recent studies have proposed training regimes that utilize the visual feedback of hand force during manipulation to improve hand function of stroke patients [19-23]. For instance, Seo et al. proposed the use of repeated practice of pinch movements coupled with visual feedback of the force direction to correct the force of the digit perpendicular to the object’s surface [19]. To reduce the excessive grip force of stroke patients, Quancy et al. proposed training that incorporates visual feedback of the patient’s actual grip force magnitude in relation to a target grip force [20]. Although these studies did not focus on patients with sensory loss, visual feedback of force information may provide greater improvement of hand function to patients with sensory impairment, because it provides additional information otherwise unavailable to the patients. Patients with sensory impairment will often grip an object with excessive or insufficient pinch pressure, or provide inappropriate force direction, because they do not receive the appropriate sensory feedback and must rely solely on visual feedback [24,25]. Feedback of force information will enable these patients to appropriately control the force necessary to manipulate objects.

To compensate for somatic sensation, however, patients with sensory loss have to concentrate on looking at the hand during movement of an object to achieve the motion. For instance, they have difficulty maintaining stability without watching their hand. Even if they are able to pick up and lift an object by looking at their hand, they drop the object once they look away from their hand. If the pinch pressure information is given as a visual cue, patients must attempt the difficult task of simultaneously looking at both their fingers and the display during manipulation. Therefore, it would be difficult for them to access additional force information through vision. If we employ a modality other than vision, such as tactile sensation, for feedback regarding pinch pressure, the patient would be able to concentrate on looking at the fingers while still receiving information about the pressure. Furthermore, to recognize force, receiving information through a similar tactile modality may be more natural than receiving this information visually.

In the present study, we hypothesized that lack of sensory feedback is a key factor in the non-use of the affected hand of stroke patients in their daily activities. We proposed a system that provides sensory feedback by transcutaneous electrical nerve stimulation (SENS) to improve manipulation capability of stroke patients with sensory loss. The system was designed to supplement sensory feedback during training to grasp or pinch objects, and to facilitate manipulation capability despite no significant recovery of haptic sensation. A stroke patient with severe sensory loss was trained to perform grasping or pinching tasks while receiving SENS, and we assessed the subsequent improvement of the patient’s manipulation capability, and long-term retention of any improvement.

**Figure 1:**
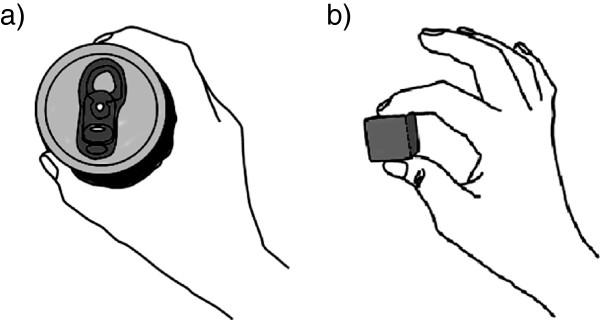
**Cylindrical grasp task and tip pinch task. a**) The fingers and thumb close and flex around the object in the cylindrical grasp task. **b**) The tip of the thumb is pressed against the tips of other fingers. In tip pinch training, the patient was asked to pinch a small cube between the tips of the thumb and index finger.

The movements practiced during training were cylindrical grasp and tip pinch (Figure 1) because the patient found it difficult to perform these motions before intervention. Tip pinch was more difficult for the patient to perform because the thumb and index finger have to oppose each other and the fingers have to apply equal force to an object. Therefore, the patient was given training first in the cylindrical grasp task (Experiment 1), and then the tip pinch task (Experiment 2). This work was partly presented in a poster at the 12th International Conference on Rehabilitation Robotics (ICORR), 2011 [26].

## Methods

### Participant

**Figure 2:**
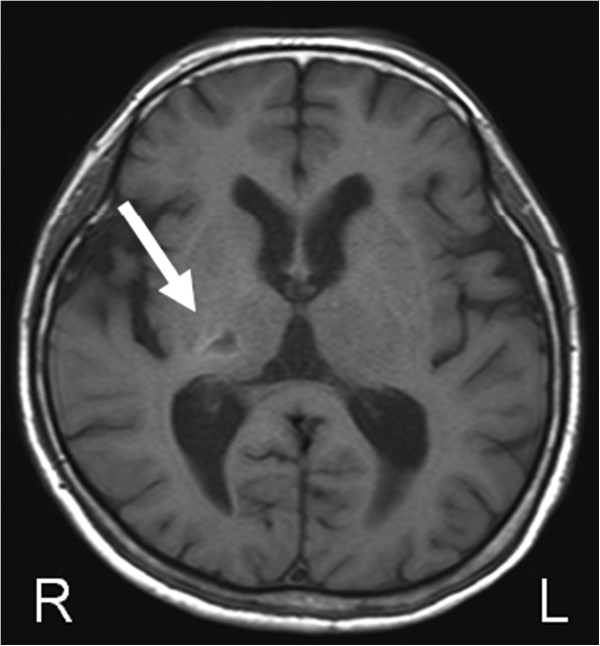
**T1-weighted magnetic resonance image of the patient’s brain.** The arrow indicates a small, low intensity area in the right thalamus.

A 66-year old female was recruited to the study. She suffered a right thalamic hemorrhage 27 months prior to the study and subsequently developed left hemiparesis (Figure 2). Her major complaint was that the affected hand was useless in daily activities, although she could move her arm and fingers. Functional assessment of upper extremity motor and sensory impairment revealed mild impairment of motor function, while her sensory deficit and manipulation deficit were severe (Table 1).

**Table 1:** Summary of functional assessments

**Assessment**	**Before experiments**	**After experiments**
Fugl-Meyer (score/total score)	60/66	60/66
Simple test for evaluating hand function (score/total score)	7/100	16/100
Nine hole peg test (time to complete)	Incomplete	Incomplete
Semmes-Weinstein monofilament test	Complete loss	Complete loss
Thumb finding test (0–3)	3	3
Moberg pickup test (time to complete)		
Eyes open	55.0 s	19.5 s
Eyes closed	Incomplete	Incomplete

The patient gave written informed consent before participating in the study, which was approved by the local ethics committee of Tokyo Bay Rehabilitation Hospital, Japan.

### Clinical assessments

**Figure 3:**
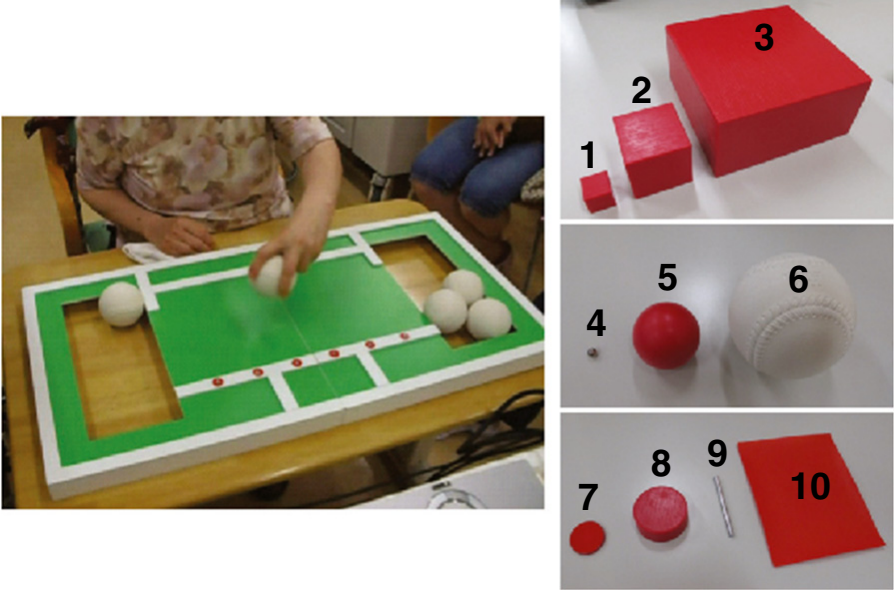
**Simple Test for Evaluating Hand Function (STEF).** The STEF, which was developed in Japan, is a test for evaluating the patient’s ability to pinch, grasp and transfer objects. The patient is required to pick up items one by one from a storage space and move them into a target space as quickly as possible. The subject performs the object-moving tests using 10 kinds of objects with different shapes and sizes, including (1) six small cubes (1.5 cm on a side), (2) six middle-sized cubes (3.5 cm on a side), (3) five large cuboids (5 cm long, 10 cm wide and 10 cm tall), (4) six small balls (0.5 cm in diameter), (5) six middle-sized balls (4 cm in diameter), (6) five large balls (7 cm in diameter), (7) seven metallic circular disks (2 cm in diameter and 0.2 cm thick), (8) six wooden circular disks (3 cm in diameter and 1 cm thick), (9) eight pins (0.3 cm in diameter and 4 cm long) and (10) six pieces of cloth (9 cm long and 7 cm wide).

To investigate whether the patient’s motor and sensory functions changed throughout the experiment, the following assessments were executed by an occupational therapist before and after conducting the experiment. Upper extremity motor function was examined using the FMA [27,28]. For the FMA, each activity received a score of 0, 1, or 2, which corresponded to no motion, partial motion, or full motion, respectively. The maximum number of points one can achieve with the FMA for upper extremity motor function is 66. To examine the patients’ manipulation capabilities, we used the Nine Hole Peg Test (NHPT) [29], Moberg Pick-up Test (MPT) with eyes open [30] and the Simple Test for Evaluating Hand Function (STEF) [31]. In the NHPT, the patient was asked to take pegs from a container, one by one, and place them into the holes on the board, as quickly as possible. Then, the patient had to remove the pegs from the holes, one by one, and place them back into the container. Scores were based on the time taken to complete the task. The MPT with eyes open assessed the ability to identify and manipulate objects. The patient was asked to pick up ten different objects one by one and place them in the pot as quickly as possible. The time taken to complete the task was measured. The STEF was developed and is used solely in Japan, and evaluates the patient’s ability to pinch, grasp, and transfer objects (Figure 3). Results of this test were standardized to the results of 1,205 healthy subjects between 3 and 90 years old, and evaluated practical use in 185 patients who suffered hand disabilities. The patient was required to pick up items one by one from a storage space and move them into a target space as quickly as possible. Each object-moving test was performed using specific numbers of a specific item, and ten types of a specific item with different shapes and sizes were used to assess the level of the manipulation capability in detail. If the patient finished each object-moving test within a specified time frame, the score of each test was calculated according to the time to finish the test. If the patient exceeded the time limit, the score of the test was 0, the minimum score. The maximum score of each object-moving test was 10, and the total maximum score for the STEF was 100. In addition, we used the Box and Block Test (BBT) [32] to assess daily improvement of manipulation capability by tip pinch task training in Experiment 2. In the BBT, the subject was required to transport blocks over the partition, and release blocks on the opposite side. The number of blocks transported to the opposite side in 1 min was recorded. Sensory function was examined using the Semmes-Weinstein monofilament test (SWMT) [29], Thumb-Finding Test (TFT) [30], and Moberg pickup test (MPT) with eyes closed. Joint position sense, vibratory perception sense, and joint motion sense of her left shoulder, elbow and wrist were also assessed. In the SWMT, a thin monofilament was pressed against the patient’s fingertips until the monofilament bent. Without any visual feedback, the patient was asked to report whether any pressure was felt. If the patient felt nothing, the next thinnest monofilament was tested. This was repeated with thicker monofilaments until the patient detected pressure. In the TFT, the affected arm was supported in front, and the patient was asked to find her thumb with her unaffected hand with her eyes closed. The score range was from 0 to 3, which corresponds to no difficulty, slight difficulty, moderate difficulty, and severe difficulty, respectively. To assess whether the patient could identify objects without visual information, the patient performed the MPT with her eyes closed. Reliability of these assessments was confirmed in previous studies [27-35].

### Sensory feedback by transcutaneous electrical nerve stimulation (SENS)

**Figure 4:**
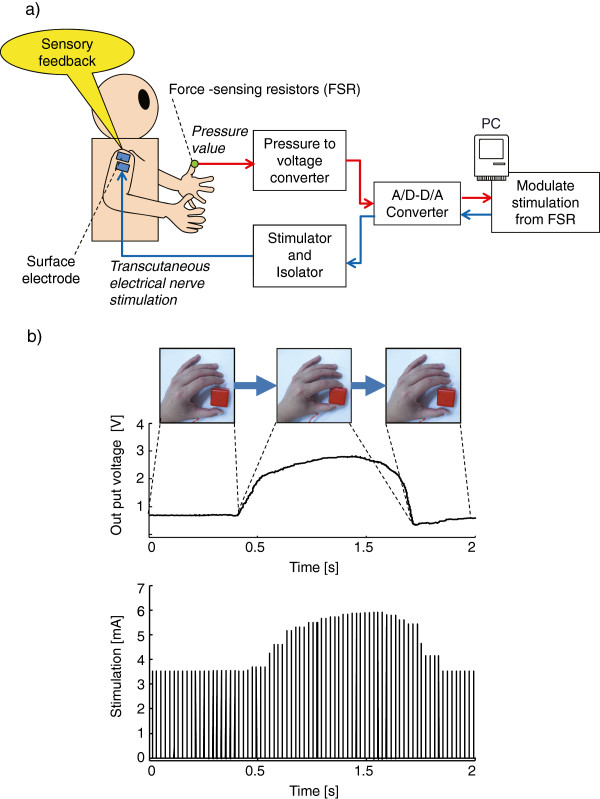
**Conceptual diagram of sensory feedback by transcutaneous electrical nerve stimulation (SENS). a**) Schematic overview of SENS. **b**) Upper panel is an example of the temporal profile of the output voltage. Lower panel represents the cutaneous stimulation sequence modulated in real time according to the output voltage shown in the upper panel.

The SENS system consisted of five major components: force-sensing resistors, a computer, a stimulator, an isolator, and surface electrodes for transcutaneous electrical nerve stimulation (Figure 4a).

Electric current pulses were delivered between two electrodes by an electric stimulator (SEN-7203, Nihon Kohden, Tokyo, Japan) and an isolator (SS-104, Nihon Kohden). Commercial electrodes (Omron Elepuls, Omron, Kyoto, Japan) were cut to customize the shape and size (roughly circular with a diameter of 3 to 5 cm). The location of electrode pads was specific to the patient. Before we fixed the electrode location in each experiment, we tested several locations where sensation was preserved, for instance, the affected shoulder, unaffected shoulder, and upper arm of the unaffected side. We asked the patient which location was most comfortable to probe the strength of stimulation. The patient chose the base of the neck on the unaffected side in Experiment 1 and both sides of the base of the neck in Experiment 2. The distance between the centers of the electrodes was approximately 5 cm.

The force-sensing resistor is a polymer thick film device with a 5.0 mm diameter active area (Standard 400 FSR, Interlink electronics, Camarillo, CA, USA) and can detect force ranging from 0.1 to 100 N. The force-sensing resistor acts as a variable resistor, with resistance decreasing in response to an increase in the force applied to the active area. A battery (9 V), resistance (10 kΩ), and the force-sensing resistor were connected in series to detect the voltage of the resistance. If no pressure is applied to the force-sensing resistor, the resistance of the force-sensing resistor reaches an infinite value and the voltage of resistance becomes approximately 0. A monotonic increase in the voltage of resistance is observed when there is a decrease in the resistance of the force-sensing resistor due to an increase in the force applied to the active area. The pressure applied to the fingertip of the patient was approximately 0 to 40 N, which corresponded to the minimum and maximum voltage values. The voltage values varied within the maximum and minimum range according to the pressure applied. Although the relationship between pressure and voltage was not linear, the output voltage indicated the features of the patient’s grasping force.

For the present clinical use of the system, the force-sensing resistor was applied to the tip of the fingers. The sensor was small enough to fit on her fingertip, and the whole surface of the sensor was secured to the fingertip with tape to stabilize the connection between the sensor and the fingertip. The pressure was detected at a sampling rate of 1 kHz and was delivered to the computer through an AD board (ADA16-32/2 (CB averaged over a 150 ms time window.

The intensity of the current was modulated according to the following equation:

(1) $$\mathrm{stim}\left(\mathrm{nT}\right)=\mathrm{stimL}\;\left(\mathrm{ip}\left(\mathrm{nT}\right)<\mathrm{ipL}\right)$$

(2) $$\begin{array}{l} \mathrm{stim}\left(\mathrm{nT}\right)=\frac{\mathrm{stimU}-\mathrm{stimL}}{\mathrm{ipU}-\mathrm{ipL}}\times \left(\mathrm{ip}\left(\mathrm{nT}\right)-\mathrm{ipL}\right)\\ \;+\mathrm{stimL}\;\left(\mathrm{ipL}<\left(\mathrm{nT}\right)<\mathrm{ipU}\right) \end{array}$$

(3) $$\mathrm{stim}\left(\mathrm{nT}\right)=\mathrm{stimU}\;\left(\mathrm{ipU}<\mathrm{ip}\left(\mathrm{nT}\right)\right)$$

where *ip*(*nT*) represents the sample at time *nT* of the averaged pressure signal, *stimL* is the lower threshold of the stimulation current (defined as the perception threshold), *stimU* is the upper threshold of the stimulation current (defined as the intensity of current that did not elicit muscle contraction and maximally 10 mA), *ipL* and *ipU* are the minimum and maximum integrated pressures observed when pinching an object, and *stim*(*nT*) is the magnification factor of the current at time *nT*. Prior to therapy each day, the patient grasped an object with minimum and maximum fingertip pressure to determine *ipL* and *ipU*. In addition, since *stimL* and *stimU* depend on individuals and vary from day to day because of skin resistance, the distance of the stimulation electrodes, and subtle difference in position of electrodes, we modulated the strength of stimulation and decided which strength was suitable for *stimL* and *stimU* each day.

In the present investigation, the actual intensity of stimulation current at time, *nT*, was calculated according to the following equation:

(4) $$\mathrm{current}\left(\mathrm{nT}\right)=\mathrm{stim}\left(\mathrm{nT}\right)f\left(\mathrm{nT}\right)$$

In equation (4), *f*(*nT*) is set as a monophasic rectangular pulse sequence at an frequency of 50 Hz and duration of 300 μs. These parameters are appropriate for activating skin sensory sensation [36,37]. The stimulation sequence was generated by an electric stimulator and applied to the electrode via an isolator. Figure 4b shows an example of the output voltage value and stimulation sequence, respectively. The stimulation sequence was modulated in real time by the amplitude of pressure.

Signal processing was performed using MATLAB 2007b (MathWorks, Natick, MA, USA).

### Tasks and intervention periods

**Figure 5:**
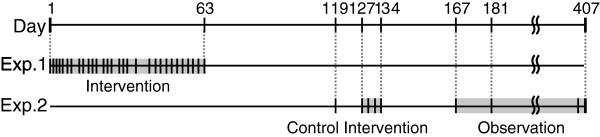
**Protocol throughout the study.** Exp. 1 and Exp. 2 represent Experiment 1 and 2, respectively. The first day of Experiment 1 was considered Day 1. Experiment 1 was conducted over 29 days during a 2-month intervention period. Experiment 2 started 56 days after the end of Experiment 1. Day 119 was set as a control day to check baseline ability, and then, there were 4 days for intervention (Day 127, 129, 132, and 134), and 5 days for observation (Day 167, 169, 181, 405, and 407).

**Figure 6:**
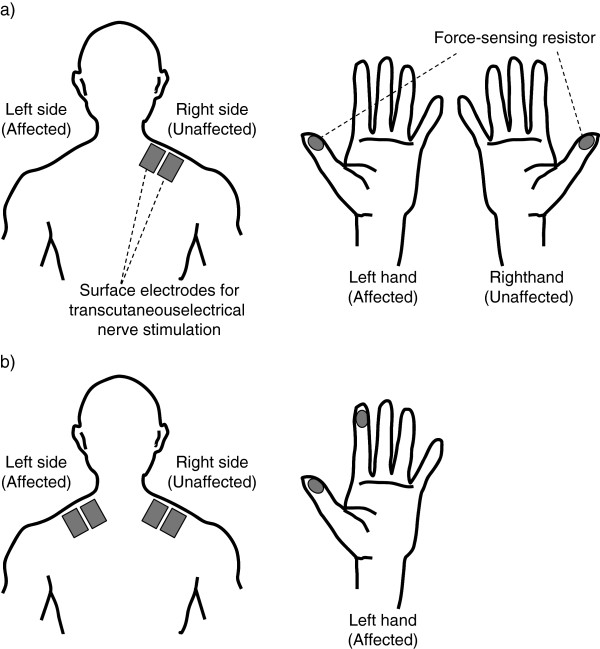
**Placement of force-sensing resistors and surface electrodes. a**) During the cylindrical grasp task, force-sensing resistors were placed on the tip of the thumb of both hands, and a pair of surface electrodes was placed on the skin at the base of the neck on the unaffected side. **b**) During the tip pinch task, force-sensing resistors were placed on the tips of the thumb and index finger of the affected hand, and two pairs of surface electrodes were placed on the skin at the base of the neck of the affected and unaffected side, respectively.

The protocol of the study is shown in Figure 5. The first day of Experiment 1 was considered Day 1. In Experiment 1, the patient was given 1 hour to train for the cylindrical grasp task per day for 29 days during a 2-month intervention period. In Experiment 1, we set a long period of training, because we were not certain how long it would take to observe improvement and when performance would plateau. The force-sensing resistors were attached to the tips of the thumb of both affected and unaffected hands. A pair of stimulation electrodes was placed on the skin at the base of the neck on the unaffected side (Figure 6a). If the pressure of both hands was fed back to different places, for instance pressure of the right hand was fed back to the right shoulder and that of the left hand was fed back to the left shoulder, then it would be difficult to compare the strength of stimulation of the affected hand to that of the unaffected hand. Therefore, both the left and right thumb pressure was fed back to the same stimulation electrodes. Any switching mechanisms between the left or right thumb (e.g., using motion sensor, or using operator’s own observation), however, may cause delay and disturb the patient. Thus, instead of having a selector switch, we asked the patient to use only one hand at a time, while we actually fed back the addition of the left and right thumb pressure to the same stimulation electrodes. The patient had no difficulty in feeling stimulation from the left and right thumb separately, and did not suffer any pain because the maximum intensity used was determined as *stimU*. The patient was able to feel stimulation when she grasped objects with either the unaffected or affected hand. First the patient would take an object with the unaffected hand to feel the stimulus pattern and intensity generated by her grasp, and then she would attempt to generate a similar stimulus pattern and intensity while performing the same task with the affected hand. During each 1-hour intervention with SENS, the patient practiced grasping items such as an ellipsoidal can, a paper cup, fabric balls, sponges and a plastic bottle, and was encouraged to rest at any time during training if she felt tired.

Experiment 2 started 56 days after the end of Experiment 1. To examine short-term and cumulative effects of training and its carry over more systematically, we established a 10-day intervention period including control and observation periods. The patient practiced pinching and lifting wooden cubes (2.5 cm per side), marbles, and buttons using the tips of the thumb and index finger of the affected hand. On the first day of Experiment 2 (Day 119), the patient received 1-hour training of the tip pinch task without SENS to assess baseline manipulation capability (control). The patient practiced the tip pinch task with SENS for 1 hour daily on Day 127, 129, 132, and 134 (intervention period). To assess long-term retention of improvement, the patient performed the tip pinch task for 1 hour per day without SENS on Day 167, 169, 181, 405 and 407 (observation period). The force-sensing resistors were attached to the tips of the thumb and the index finger on the affected hand. Two pairs of stimulation electrodes were placed on the skin on either side of the base of the neck (Figure 6b). The stimulation based on the thumb tip pressure was provided to the affected side of the neck, while that based on index fingertip pressure was provided to the unaffected side.

Besides, during the period over which Experiments 1 and 2 were performed, the patient also continued her conventional physical and occupational rehabilitation program. The rehabilitation program was conducted for 2 hours per day for 3 days a week and did not change throughout the study. To confirm that conventional physical and occupational therapy did not improve her manipulation capability further, we compared results of the STEF conducted on the first day of intervention (Day 1), before the patient first experienced SENS, with that of 1 week prior to Day 1. STEF scores were 6 and 7, respectively, showing that the last 3 days of conventional therapy did not improve manipulation capability, assessed by the STEF.

### Assessment for each training task

#### Experiment 1: cylindrical grasp task

To assess the fluctuation in grip force during cylindrical grasp, we asked the patient to grasp and lift a 370 g can for approximately 10 s. We measured the output voltage values during this assessment task under two conditions: 1) before training – at this time the patient was assessed on her ability to perform the cylindrical grasp task without receiving SENS, and 2) after training – the patient trained while receiving SENS and was later assessed while also receiving SENS. Voltage was sampled at a rate of 50 Hz and was measured using a data acquisition system (PowerLab 16/30, AD Instruments, Sydney, Australia).

We defined the average rate of change in voltage values as the *fluctuation index,* which indicates the fluctuation in force during can grasp assessment. The *fluctuation index* was calculated from the signals between the onset (the output voltage exceeds zero) and offset (the voltage becomes zero) of force, according to the following equations:

(5) $$\mathrm{\Delta v}\left(\mathrm{mT}\right)=s\cdot \left(v\left(\mathrm{mT}\right)-v\left(\left(m-1\right)T\right)\right)$$

(6) $$\mathrm{fluctuation}\;\mathrm{index}\;=\frac{1}{k-1}\sum_{m-1}^{k-1}\left|\mathrm{\Delta v}\left(\mathrm{mT}\right)\right|$$

In equations (5) and (6), *v* is the output voltage value, *s* is the sampling rate, Δ*v*(*mT*) is the rate of change at time *mT*, and *k* is the total number sampling points. For comparison with healthy function, the *fluctuation index* of the can grasp with the patient’s unaffected hand was measured and averaged across 11 assessment trials.

#### Experiment 2: tip pinch task

To assess the patient’s ability to perform the tip pinch task, we asked the patient to pinch and lift a wooden cube of side 2.5 cm for approximately 10 s. During this pinching and lifting task, we measured the output voltage values of the thumb and index finger. This assessment was done on the first day of the intervention period for this task (Day 127) without SENS before training, and then immediately after SENS was applied. The assessment was done again on the third day of the observation period (Day 181) without SENS before training. For comparison, the same assessment was completed for the unaffected thumb and index finger.

The Box and Block Test (BBT) was also used to assess the patient’s manipulation capability. During the intervention period, the patient always received SENS while training and the BBT was conducted under four conditions. The patient was assessed while not receiving SENS, both before training and after 60 min training. She was also assessed while receiving SENS, after both 30 and 60 min training. During the control and observation period, the BBT was assessed at three times during which the patient was not receiving SENS: 1) before training; 2) after 30 min training; and 3) after 60 min training.

## Results

### Clinical assessment of motor and sensory function

Table 1 summarizes the results of the clinical assessment of the patient before and after the experiments. The FMA score for upper extremity motor function was 60 both before and after the experiments. This indicates that the patient’s upper extremity motor impairment was mild even before the experiments. She was unable to detect any sensation in the left fingertips even with the largest monofilament (1.142 mm in diameter) in the SWMT, and was unable to do so even after the experiments. The patient’s TFT score was 3, indicating that she was unable to find her left thumb before and after the experiments. The patient was not able to finish the MPT with her eyes closed. In addition, her joint position sense, vibratory perception sense, and joint motion sense of her left shoulder, elbow and wrist were found to be completely or severely lost both before and after the experiments. These results indicate that she had neither superficial sensation nor proprioception in the left hand or fingers before the experiments, and that her sensation itself was not improved by the experiments.

**Figure 7:**
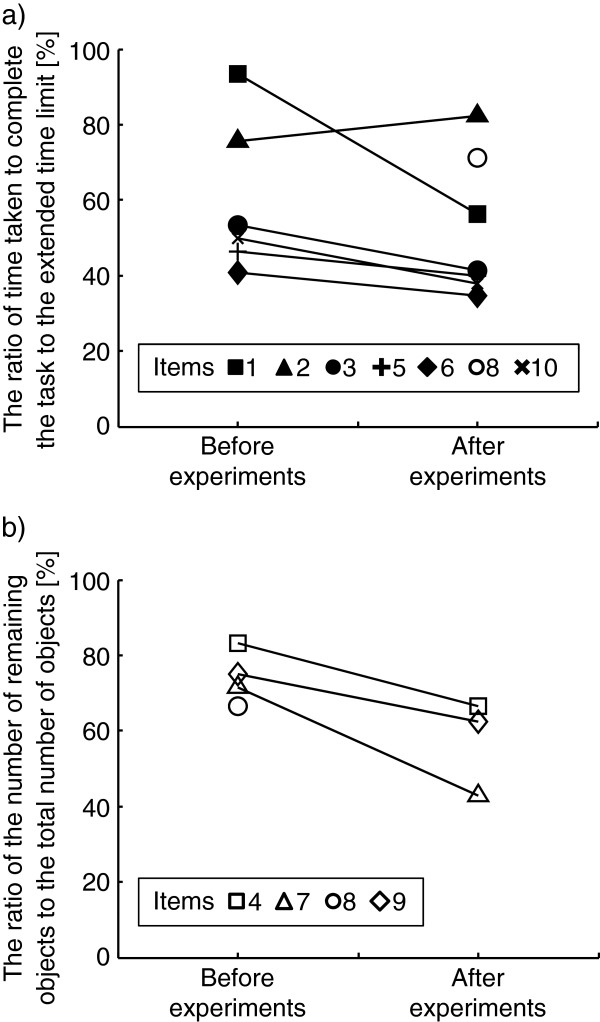
**Improvement in STEF.** Comparison of STEF results before Experiment 1 and after Experiment 2. The item numbers in the figures correspond to those in Figure 3. **a**) The time taken to complete the task was divided by the extended time limit, which was 1.5 × longer than the original time limit of each object-moving task. **b**) The number of objects left over after the extended time limit was divided by the total number of objects in each task.

Before conducting the experiments, the patient’s total STEF score was only 7 out of 100. After completing the experiments, her STEF score had improved to 16. Figure 7 shows the detailed results of the STEF, and each item corresponds to the item in Figure 3. Figure 7a shows the ratio of time taken to complete the task to the time 1.5 × longer than the original time limit of each task. The patient had difficulty completing the STEF before the experiments, but to assess her manipulation capability precisely, she was asked to continue each task until the time exceeded the extended time limit, which was 1.5 × longer than the original time limit. Except for item 2, the time taken to complete the object-moving task decreased after training with SENS compared with before training. Especially for item 8, the patient could not complete the task within the extended time limit before the experiments, but was able to finish it after the experiments. Figure 7b shows the ratio of the number of objects that the patient could not move within the extended time limit, which was 1.5 × longer than the original time limit to the total number of objects in each task. The number of remaining objects decreased after the experiments. Before conducting the experiments, the time to finish the MPT with eyes open was 55.0 s, and the patient was unable to insert any pegs in the NHPT. After completing the experiments, the time of MPT with eyes open was reduced to 19.5 s. The patient was, however, still unable to insert any pegs in the NHPT. To insert a peg in the NHPT, the patient had to re-grasp, or change the direction of a small, thin peg in her hand. Thus, this test required the combined motion of pinching and re-grasping or dexterous manipulation, and it was difficult for her even after intervention with SENS. Taken together, the results suggest that before the experiments the patient had much difficulty in manipulating objects, even though her motor impairment measured by FMA was mild, and that her manipulation capability was improved by training with SENS despite her sensory function not recovering.

### Experiment 1: cylindrical grasp task

**Figure 8:**
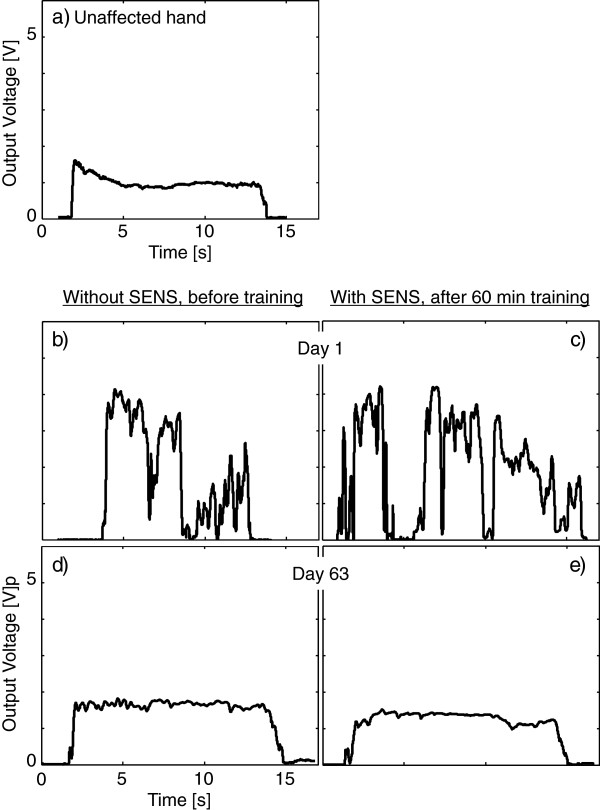
**Examples of fingertip force temporal profiles in Experiment 1 (A can grasp assessment task).** The output voltage values that correlate with fingertip force during a can grasp task were plotted against time. **a**) The output voltage value of the unaffected hand. **b**), **c**) The output voltage value of the affected hand on Day 1. **d**), **e**) The output voltage values of the affected hand on Day 63. **b**), **d**) Values assessed without SENS before training, and **c**), **e**) values assessed with SENS after training.

Before the training with SENS, we measured the ability of the unaffected hand to grasp and lift an object. Figure 8a shows the output voltage value of the unaffected thumb during the can grasp assessment task. The patient was able to maintain a stable pressure during the assessment task when using the unaffected hand. The voltage value reached a maximum value when the patient lifted up the can (for around 2 s) and gradually decreased thereafter.

Figure 8b shows the output voltage values of the affected thumb during a can grasp task assessed without SENS before training on Day 1. The voltage value was not stable and the patient dropped the can at about 9–11 s. Figure 8c indicates the output voltage values of the affected thumb assessed with SENS after 1-hour grasp training on Day 1. Even after training, the patient still dropped or nearly dropped the can at around 2, 5, 7 and 10 s. These results suggest that, on Day 1, the grip force of the affected hand were unstable and the fluctuation in grip force was not reduced even after training.

We investigated the long-term improvement effects of the intervention. Figure 8d shows the output voltage values of the affected thumb during the can grasp task assessed without SENS before training on Day 63. Figure 8e indicates those with SENS after training on Day 63. The fluctuation of the voltage value before training as well as after the training was smaller than that on Day 1.

**Figure 9:**
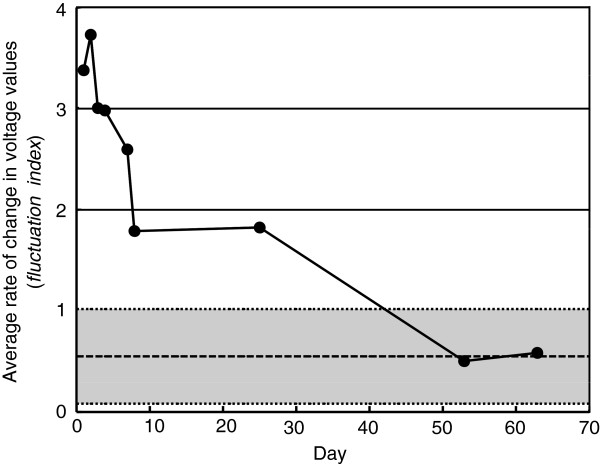
**The change of *fluctuation index.*** The *fluctuation index* of the affected hand during the can grasp task assessed with SENS after training, plotted against days from the beginning of Experiment 1. The dashed line and gray zone are average and average plus/minus two standard deviations of *fluctuation index* of the unaffected hand, respectively.

Figure 9 shows the *fluctuation index*, or stability, of the affected hand during the can grasp task assessed with SENS after 1-hour training. The dashed line and gray zone represent the average and the average plus or minus two standard deviations of the *fluctuation index* of the unaffected hand measured on Day 1, respectively. The patient was able to maintain a more stable pressure on Day 53 compared with Day 1. The *fluctuation index* decreased as the training progressed, and those on Day 53 and Day 63 were almost the same as those observed in the unaffected hand.

### Experiment 2: tip pinch task

#### Fingertip force during pinching and lifting task

**Figure 10:**
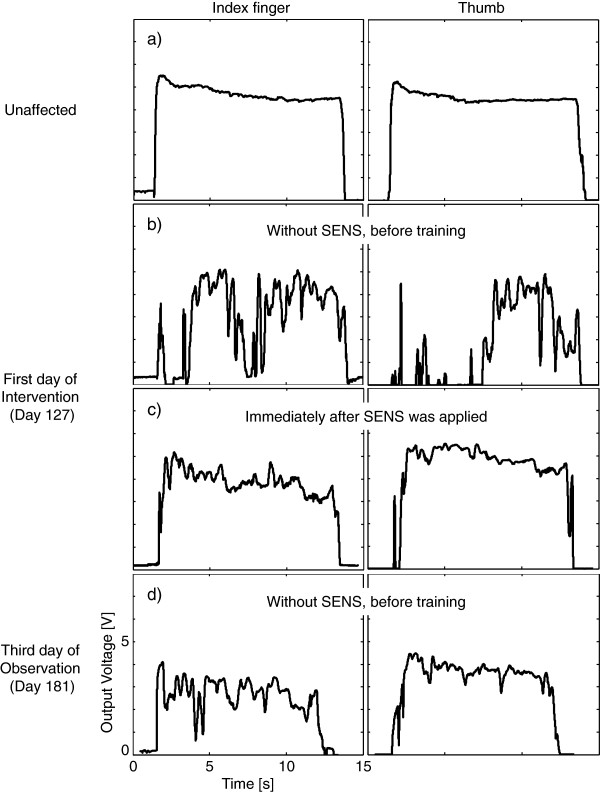
**Examples of tip pinch force profiles in Experiment 2 (Tip pinch task).** The output voltage values during a cube pinching and lifting task were plotted against time. **a**) The output voltage values of the unaffected hand, **b**) of the affected hand assessed without SENS before training, **c**) of the affected hand immediately after SENS was applied on the first day of intervention (Day 127), and **d**) of the affected hand assessed without SENS before training on the third day of observation (Day 181). The left column shows the output voltage value of the index finger, and the right column shows that of the thumb.

Figure 10 shows the output voltage values of the index finger and thumb. The patient was able to maintain a stable pressure during the task with the unaffected hand (Figure 10a). The voltage value reached a maximum value when the patient lifted up the object (for around 1 s) and it gradually decreased thereafter. Before training on the first day of intervention (Day 127), the voltage values of both the thumb and index finger were unstable during the tip pinch task (Figure 10b). The patient could not properly contact the thumb with the surface of the cube and she nearly dropped the cube at around 2–4 s. In contrast, when SENS was applied, both the thumb and index finger contacted the block properly and the stability of the fingertip pressure was improved (Figure 10c). Even in the third day of observation period (Day 181) 47 days after the end of intervention (Day 134), the thumb and index finger were still able to properly contact the surface of the cube compared with the first day before training (Figure 10d).

#### Box and Block Test (BBT)

**Figure 11:**
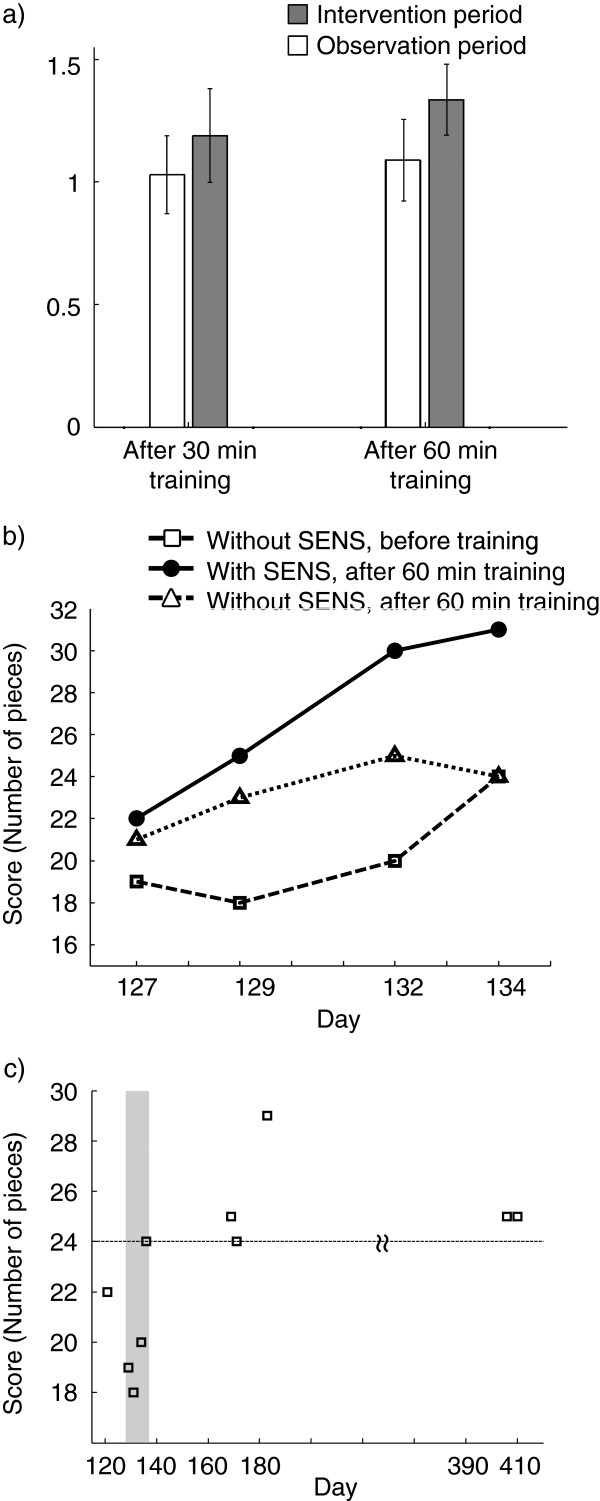
**The scores of the Box and Block Test (BBT). a**) Improvement of the BBT score. That is, the BBT score after training divided by that before training on the same day. The BBT test after training was conducted with SENS during the intervention period. The gray bar is the improvement rate for the intervention period, and the white bar is that for the observation period. The left bars show the result after 30 min training, and right bars show that after 60 min training. **b**) The BBT scores on each intervention day. Open squares denote the scores of BBT assessed without SENS before training. Filled circles denote BBT scores assessed with SENS after 60 min training. Open triangles denote BBT scores assessed without SENS after 60 min training. **c**) The score of BBT assessed without SENS before training throughout the control, the intervention and the observation period, plotted against the interval scale of time. The gray zone indicates the intervention period. To evaluate whether the patient was able to maintain the improved manipulation capability or not, BBT score assessed without SENS after 60 min training on the last day of intervention (Day 134) was marked by the broken line.

To evaluate the effectiveness of the combination of task-specific training of pinching various objects and SENS on manipulation capability, we compared the daily improvement rate of the BBT during the intervention period (training with SENS) with that during the observation period (training without SENS). The improvement rate of the BBT score was calculated by dividing the BBT score after training by that before training on the same day. The BBT after training was conducted with SENS during the intervention period. The BBT score improvement rate after 30 and 60 minutes of training was averaged over the 4 days during the intervention period, as well as over 5 days during the observation period (Figure 11a). Although improvement of score in the observation period indicated an effect of repetition of the BBT itself, the average improvement rate after both 30 and 60 minutes training was higher in the intervention period compared with the observation period. The results suggest that the short-term improvement effect of task-specific training was enhanced by SENS.

During the intervention period, we investigated the effect of training with SENS on the patient’s manipulation capability. The BBT score assessed with SENS after 60 min training was increased compared with that assessed without SENS before training each day (Figure 11b). Although the score assessed without SENS after 60 min training was lower than the score assessed with SENS after 60 min training, it was still higher than that assessed without SENS before training. In addition, the score before training gradually increased through the intervention period, suggesting a long-term training effect. We also investigated the long-term retention of the improvement in the patient’s manipulation capability. Figure 11c shows the BBT score assessed without SENS before training throughout the control, intervention and observation periods, plotted against the interval scale of time. Throughout the observation period, the patient maintained an equal or higher BBT score compared with that at the end of the intervention period.

#### Feasibility of SENS

Finally, no adverse events occurred during the experiments. The patient did not experience much difficulty in executing the tasks and appeared to enjoy the training with SENS. Thus, the feasibility of task-specific training with SENS was confirmed.

## Discussion

This study proposes a novel rehabilitation technique for stroke patients using transcutaneous electrical nerve stimulation. The technique, which we call sensory feedback by transcutaneous electrical nerve stimulation (SENS), compensates for the lost pressure sensation of the fingertips. We conducted a clinical case study of a stroke patient with severe sensory loss, and confirmed the feasibility of the proposed system.

Cylindrical grasp training with SENS gradually stabilizes the grip force. After a 2-month intervention period, the patient was able to maintain a stable fingertip pressure during the grasp task, even without SENS. The tip pinch task, however, was still difficult for her without SENS, and the thumb and index finger did not contact the surface of the object properly. When the patient executed the tip pinch task with SENS, the pinch pressure immediately stabilized. After the patient practiced the tip pinch with SENS, her manipulation capability assessed by the BBT score was also improved even after SENS was removed, for at least 7 months after the intervention period. Before the patient started training with SENS, she continued a conventional physical and occupational rehabilitation program for 3 days a week for 21 months after the recovery phase. However, her manipulation capability was largely unchanged with conventional therapies. In general, the recovery phase is from several weeks to 6 months after stroke onset, and survivors continue their rehabilitation in the chronic phase to maintain any improved function acquired during the recovery phase. Therefore, in this study we assumed that the improvement of manipulation capability of the patient was due to training with SENS, and not due to conventional therapies.

The fact that the patient was able to achieve tip pinch immediately after SENS was applied demonstrates the importance of sensory feedback information in dexterous manipulation of objects. The role of sensory function on motor control has been investigated in deafferented patients and by blocking sensory input during motion by ischemia or anesthesia [38-41]. Though the simple output of muscle power or joint motion is possible without proprioceptive and haptic feedback, the loss of sensory feedback causes incoordination between multiple joints, reduces accuracy in motion direction and preshaping of the hand, and interrupts adaptation to environment during motion. The patient in this study was able to achieve simple hand motions, such as flexion and extension of joints, as assessed by FMA. Nevertheless, the lack of sensory feedback caused poor performance in manipulation tasks, which is in accordance with previous studies. Biological movement consists of both feedforward and feedback components, but dexterity likely depends mainly on feedback control [42-44]. A dependency of dexterity on feedback control was supported by the present study which demonstrated that the feedback control loop was improved by sensory feedback from SENS, and this improved feedback control in turn provided immediate improvement of manipulation. Similar improvement of manipulation has been reported for sensory feedback in studies of the myoelectric prosthetic hand which can provide touch sensation of the fingers or palm [45-48]. For instance, a user of this prosthetic hand reduced contact force of the fingers during grasping when receiving force feedback information through vibrotactile stimulation [47]. The strength of the vibrotactile stimulation was correlated with the hand force. Similarly, Stepp et al. reported that virtual object manipulation by healthy subjects was improved more when provided with both visual and vibrotactile feedback compared with visual feedback alone [48]. The patient in this study had already received extensive physical and occupational therapy prior to experiments. However, her manipulation capabilities were not significantly changed by these conventional processes. The patient might have depended excessively on visual feedback and applied too much feedback gain, causing unstable force control during object manipulation. Our results suggested that force feedback in addition to visual feedback is crucial to improve manipulation capabilities, and the proposed SENS system might be useful for sensory assistance. An immediate effect of SENS was not observed during cylindrical grasp training. This could possibly be because the patient had learned how to use feedback information from SENS, and had reconstructed a feedback loop using SENS at this initial phase of training.

The improvement and retention of the patient’s manipulation capability compared with training without SENS suggests that the patient learned how to manipulate objects through training with SENS. Because sensory feedback was not available during the observation period, the patient had to control force using the learned feedforward control instead of the feedback control strategy using SENS information. To produce desired force in a feedforward manner, an internal model must compute the necessary motor command before the movement can be initiated. Again, sensory feedback plays an important role in learning such mapping between motor command and force [49]. Several previous studies demonstrate impairment of motor learning due to a lack of sensory feedback. For instance, monkeys with a lesion of the hand area in the somatosensory cortex of one hemisphere of the brain had severe difficulty in learning the new skills with the hand contralateral to the ablated somatosensory cortex [50]. Patients deprived of limb proprioception because of large-fiber sensory neuropathy experience great difficulty in reaching a target only 10 centimeters from their hand [51]. This suggests the possibility that the deprivation of sensory input not only prevents learning of new skills but also causes the degradation of already existing motor skill, or internal models. In the current study, the patient might have developed a new internal model or might have recalibrated an existing internal model by training with SENS feedback. Subsequently, the patient might have become able to generate desired force without sensory feedback, through recovery of feedforward control. With regard to the improvement in manipulation capability, there is also the possibility that training with SENS allowed the subject to use remaining sensory feedback in an enhanced way, which could not be detected by standard clinical scales.

The improvement rate of the BBT score was higher when the patient practiced the tip pinch task of various objects with SENS. In rehabilitation for stroke patients, it is widely recognized that task-specific practice is important for improvement of manipulation capability, in addition to training for general improvement of muscle strength, range of motion, etc. For instance, constraint induced therapy (CIMT) is known to be an effective rehabilitation therapy that improves upper extremity function in stroke through task-specific training [52]. Even after the training without SENS, the BBT score increased, supporting the importance of also using task-specific training for treatment of stroke patients with sensory loss. The improvement in the BBT score was greater when SENS was applied during training, indicating that task-specific training of pinching various objects in combination with sensory feedback enhanced motor learning of the patient. Although manipulation capability gradually improved over the training with SENS, sensory function did not recover. Regaining lost sensory pathways and recovering sensation seems to be difficult [6]. Therefore, training focusing on motor learning, rather than recovery of sensation, would be more effective for severe sensory loss patients to recover daily use of the affected hand. Kottke et al. [53] defined an engram as a sequence of motor commands for muscles and described that thousands of repetitive motions could enhance the engram. The repetitive grasping and pinching training using various objects in this study contributed to the improvement in the patient’s manipulation capability.

Because the STEF was conducted as one of the clinical assessments before Experiment 1 and after Experiment 2, and the objects used in the STEF were not used in the training with SENS, the improvement of STEF scores was considered the result of generalization of acquired manipulation capability to different objects. Additionally, the patient reported that she used her affected hand more often in daily activities. For example, she is now holding a showerhead with her affected hand while shampooing her hair, and is holding food ingredients with her affected hand when she cuts it with a knife. These findings indicate that acquired manipulation capability was generalized to tasks outside those in the current experiments.

We choose transcutaneous electrical nerve stimulation for sensory feedback because electrical stimulation is widely used as a treatment in hospitals. Other benefits of electrical stimulation are as follows [54,55]. First, it is possible to make the whole system smaller and lighter so that patients can use the system not only at hospital but also at their home. Second, it does not require consideration of the resonance characteristic of the stimulator, which is a problem in mechanical stimulation system such as vibrotactile or pressure stimulation. Third, since patients need to wear only thin pads for electrical stimulation, limitation for body movement is reduced compared with other stimulation methods. Several studies have already tested the use of other modalities to provide sensory feedback information. Visual feedback of hand force was shown to contribute to force control when stroke patients manipulate objects [19-23], and auditory feedback of finger tactile information was shown to contribute to the recovery of touch sensation after neural repair [56-58]. These results indicate that other feedback methods, such as mechanical, visual, and sound stimulation, may also help improve the manipulation capability of patients with severe sensory loss, and additional work is required to investigate which feedback method is more efficient.

SENS can also be applied to other parts of the body. For instance, we can set force-sensing resistors on the soles of the feet and provide contact pressure information during walking. Nor is SENS limited to stroke patients but is also applicable to a wide range of patients with sensory disturbances.

Limitations of this study include the small sample size without control, lack of kinematic analysis and lack of investigation regarding the real amount of use of the affected hand in daily activities. Furthermore, most stroke patients have both motor and sensory deficit, and the current study did not investigate whether SENS could be applied to the entire stroke patient population. Therefore, research in a larger cohort of patients is required to properly evaluate the efficacy of the system. Furthermore, the present system is so large that it can be used only in a laboratory or hospital setting. In future studies, we aim to increase the number of participants recruited, conduct long-term follow-up, use appropriate clinical assessment measures to evaluate the amount of use of the affected hand, and develop a smaller system for use at home.

## Conclusions

In this study, we developed a system that provides sensory feedback by transcutaneous electrical nerve stimulation (SENS) for stroke patients with sensory loss. A stroke patient with severe sensory loss was trained to perform cylindrical grasp and tip pinch tasks with SENS, and the feasibility of SENS was assessed. Results demonstrated that the patient’s manipulation capability was improved through training with SENS and she maintained the manipulation capability even after SENS was removed, despite there being no recovery of sensation. We conclude that because SENS is very simple, it may be a valuable contribution to the rehabilitation of patients with sensory loss.

## Competing interests

The authors declare that they have no competing interests.

## Authors’ contributions

The overall design of the experiments was agreed on by all the authors after extensive discussions. KK developed the proposed system, carried out the all experiments, analyzed the data and drafted the manuscript. YO, a medical doctor, contributed to conception and design, selected the stroke patient, carried out interpretation of the data, and revised the manuscript. KT developed the system and made substantial contribution to acquisition of the data. SS, an occupational therapist, evaluated the clinical scores. JU contributed to development of the system, and revised the manuscript. KK and ML revised the manuscript. RO was involved in conception and design of the study, carried out interpretation of the data and revision of the manuscript. All authors read and approved the manuscript.
